# School Refusal or Truancy? A Qualitative Study of Misconceptions Among School Personnel About Absenteeism of Children From Immigrant Families

**DOI:** 10.3389/fpsyt.2020.00202

**Published:** 2020-03-20

**Authors:** Robin Martin, Jean Pierre Benoit, Marie Rose Moro, Laelia Benoit

**Affiliations:** ^1^Maison des Adolescents de Saint-Denis, Saint-Denis Hospital, Saint Denis, France; ^2^Maison des Adolescents—Maison de Solenn, Hôpital Cochin, APHP, Paris, France; ^3^University of Paris, Medical School, Faculty of Psychology, PCPP, Boulogne-Billancourt, France; ^4^Center for Research in Epidemiology and Population Health (CESP), Paris-Sud and UVSQ Medical Schools, French National Institute of Health and Medical Research (Inserm), Team DevPsy, Villejuif, France

**Keywords:** school refusal, truancy, teacher, school personnel, immigrant youth, minorities, discrimination, school absenteeism

## Abstract

**Background:**

School refusal is a form of school attendance problem (SAP) distinct from truancy, school withdrawal, and school exclusion; it requires specific mental health care. Schools' identification and referral to care of school refusers depends on school personnel's interpretation of the reasons for absences. Because cultural factors can induce misunderstanding of the young people's behavior and of their parents' attitudes toward school attendance, school personnel can have difficulty understanding these reasons for children with transcultural backgrounds (migrants or children of migrants). The aim of this study was to explore the experiences and opinions of school personnel, mainly teachers, related to school refusal among these students.

**Methods:**

Grounded theory methodology was used to conduct 52 qualitative interviews of school personnel in two regions of France. Their daily practices with students presenting with school refusal were addressed in general (i.e., in response to absence of all youth) and in transcultural contexts (i.e., absence of migrant children or children of migrants). This study analyzed the interviews of the 30 participants who reported working with students from transcultural backgrounds.

**Results:**

Many school personnel reported experiencing difficulties, ambivalence, and destabilizing feelings in situations involving immigrant families whose school culture differed from their own. Talking about culture appeared to be taboo for most participants. These situations challenged the participants' usual strategies and forced them to devise new ones to deal with these young people and their families. Although some personnel were at risk of developing exclusionary attitudes, others dealt with school refusal with both commitment and creativity.

**Conclusion:**

The tensions experienced by these participants reveal contradictions between the French universalist ideology and the reality of daily life in schools becoming increasingly multicultural. School personnel's attitudes toward children with transcultural backgrounds presenting with school refusal can affect children's access to care and shape social inequalities. Further research should develop, implement, and assess interventions including transcultural training of school personnel, improved use of interpreters at school for migrant families, and the addition of a transcultural dimension to SAP assessment scales, especially for school refusal.

## Introduction

School refusal is a frequent reason for consultation at child mental health services; it is thought to concern about 1% of pupils and 5% of consultations in preadolescence and adolescence (1) and can be associated with comorbidities such as anxiety or depression (2). It was first described in the literature as “school phobia” in 1941 (3). Over the past 60 years, authors have studied its diverse causes and multiple consequences, including the worrisome prognosis associated with extended absenteeism (4) and the other disorders for which it is a risk factor (5). They have also analyzed the role of the family context (6) and the functions of school refusal (7, 8). The emerging international consensual definition of school refusal, which distinguishes this form of school attendance problem (SAP) from others, including truancy, school withdrawal, and school exclusion, relies on four criteria (9): “(1) a young person is reluctant or refuses to attend school, in conjunction with emotional distress that is temporal and indicative of aversion to attendance (e.g., excessive fearfulness, temper tantrums, unhappiness, unexplained physical symptoms) or emotional distress that is chronic and hindering attendance (e.g., depressive affect; sleep problems), usually but not necessarily manifest in absence (e.g., late arrivals; missing whole school days; missing consecutive weeks, months, or years); and (2) the young person does not try to hide associated absence from their parents (e.g., they are at home and the parents are aware of this), and if they previously hid absence then they stopped doing so once the absence was discovered; and (3) the young person does not display severe antisocial behavior, beyond resistance to parental attempts to get them to school; and (4) the parents have made reasonable efforts, currently or at an earlier stage in the history of the problem, to secure attendance at school, and/or the parents express their intention for their child to attend school full-time.” [(9), p.15].

According to Bools and colleagues, the classification of SAPs is difficult because of the need to determine whether parents have put enough pressure on the child to go to school (10). In this conception, the existence of reasonable parental efforts to secure school attendance is considered evidence that the problem is attributable to school refusal instead of truancy. Indeed, according to Heyne et al. “Truancy is said to occur when (1) a young person is absent from school for a whole day or part of the day, or they are at school but absent from the proper location (e.g., in the school-yard rather than in class); and (2) the absence occurs without the permission of school authorities; and (3) the young person typically tries to conceal the absence from their parents.” [(9), p.16].

Nevertheless, Heyne et al. stress that a young person may display all the defining features of school refusal except that his parents have not made a “reasonable effort” to get him to school (9), which the authors define as. “attempts to address the problem, beyond the parent simply expressing to the child their desire that the child attend school. These efforts could include getting the child out of bed or into a mode of transport to go to school, contacting school staff because of nonattendance, and attending meetings aimed at addressing the problem. We acknowledge that in families with two parents, the parents may vary in their efforts to get their child to school, perhaps because of differences in parenting style or self-efficacy.” [(9), p.16].

Although this definition of parental “reasonable efforts” already takes different parenting styles into account, it still overlooks cultural factors that can cause misunderstanding of the young people's behavior and of their parents' attitudes toward school attendance. As Kearney pointed out, “cross-cultural aspects of school absenteeism and school refusal behavior remain in need of greater exploration and explication (5).” Nevertheless, information on the transcultural dimensions of school refusal remains scarce in current educational and psychiatric research (11–13). This omission of the transcultural dimension in research is reflected in guidelines for school personnel, which ignore this issue (14).

Studies have underlined the higher prevalence of absenteeism among ethnic minorities (15) and noted that SAPS among children of immigrants may be a manifestation of systemic discrimination, which could be interpreted as school exclusion (16). Bourdieu and Passeron, in the early 1960s, theorized about the role of school systems in the reproduction of social inequalities (17). They showed that students' objective probabilities of academic success depend on their cultural capital and especially on a school culture congruent with that of its personnel. This sociological perspective has become widespread in France, influencing school policies as well as school personnel, who are aware that these factors impair their students' chances of success. This awareness does not prevent them, however, from experiencing a sense of mismatch in their work with families that do not share their school culture—including immigrant families.

Although French data protection laws make it hard to collect information about minorities and migrants, one of the rare reports authorized on this topic underlined the general difficulties of academic achievement for immigrant youths (18), even beyond school attendance problems. The school refusal behavior of some adolescent immigrants is sometimes an expression of their anxiety about academic achievement (11). Relevantly, Moro has described adolescence—the age group corresponding to middle school and high school—as a period of vulnerability, especially combined with the migration-specific difficulties with which these youth and their families must cope (19). However, sociological and pedagogical studies have addressed children's transcultural backgrounds only in relation to school topics such as literacy (20), underperformance at school, discipline, and interpersonal conflicts (21). To the best of our knowledge, exploration of the attitudes and representations of immigrant parents in relation to school attendance problems is sparse (22).

One hypothesis among the many potential explanations for the visible lack of reasonable parental efforts in what is labeled truancy is the existence of cultural and social differences between the parents and the school. Evidence supporting this hypothesis might thus underline the limitations of the current classification system, including the major risk of misdiagnosis it poses for immigrant youths whose behavior might be incorrectly labeled as truancy rather than school refusal. We suggest that socio-cultural factors must be studied in the definition of school refusal, given their potential influence on the behavior of both youths and their parents and on the understanding of the personnel in both schools and health care facilities. The aim of this study was thus to explore the experiences and opinions of school personnel, mainly teachers, on the topic of school refusal among students from migrant families. To our knowledge, this study is the first to focus on school personnel's understanding of cultural factors that can shape the care pathway of adolescents with transcultural backgrounds.

## Methods

### Ethics Evaluation

This qualitative study, which followed the COREQ guidelines (23), was approved by the competent institutional review board, the INSERM Ethics Evaluation Committee (IRB00003888).

### Choice of Methodology

This research applied Grounded Theory (GT) methodology. First used in an ethnographic study of hospital patients who were dying, GT has been a standard (with many variants) methodology for social sciences research since the 1980s (24). It is a general methodology, a way of thinking about, collecting, analyzing, and conceptualizing data. It uses inductive reasoning, in contrast to hypothetico-deductive models, to construct theories through systematic gathering and analysis of data. GT also links individual and subjective experience to social processes, by focusing on themes that represent phenomena, interactions, and their consequences. The choice of GT was justified by our research question (i.e. the opinions and experiences of school personnel on the topic of school refusal among students from migrant families) for which the existing literature is extremely sparse. Moreover, other inductive qualitative methods confining the analysis to the individual phenomenological level (such as Interpretative Phenomenological Analysis), would disregard two major aspects of the question we are studying: face-to-face interactions (microsociology) and daily practices of groups (mesosociology). Thus, the application of GT enables us to interpret the results in the light of several concepts from the fields of sociology and anthropology (for further details, see the *Discussion* section).

### Inclusion

As in other inductive methods, we did not need to define an exact number of respondents before the research began. Purposive sampling (25) was used to recruit a nonprobability sample population, based on subjective criteria related to the study's goals; this method is very common in qualitative studies (which have goals different from those of quantitative studies and for which representativeness is not a criterion). In the Paris area, personnel at two middle schools (*collèges*) participated, and in Bourgogne Franche-Comté, participants came from one urban high school and two middle schools, one urban and one rural. These were all public schools, which 83% of pupils in France attend (26). These schools, of different sizes, in different geographical areas, and serving a broad range of socioeconomic groups, were selected to maximize the global heterogeneity of the sample. School personnel included teachers, principals and assistant principals, educational assistants, guidance counselors, and school doctors and nurses. Interviews were also conducted with doctors in the local education authorities and in one schooling association (that provides teaching services at home or in hospitals).

In interviews, we specifically used the standard definition of school refusal by Berg (9, 27), which includes: a) reluctance or refusal to attend school, often leading to prolonged absences, b) staying at home during school hours with parents' knowledge rather than concealing the problem from parents, c) experience of emotional distress at the prospect of attending school (somatic complaints, anxiety, and unhappiness), d) absence of severe antisocial behavior, and e) parental efforts to secure their child's attendance at school. We decided not to consider criterion (e), because, in transcultural contexts, school personnel might have even more difficulty in determining whether parents made sufficient “reasonable efforts” to get the child to go to school than in intracultural contexts (10).

### Participants

All 52 participants interviewed were asked if they worked with students with transcultural backgrounds (i.e., students who were first- or second-generation migrants, that is, migrant children or the children of migrants). Only 30 responded affirmatively that they taught or otherwise dealt with such students. These participants' understanding of these students' SAPs were discussed in their interviews, in light of the Berg criteria for school refusal (except criterion e, as stated above). The interviews of these 30 participants are analyzed here. Their characteristics are summarized in **Table 1**.

**Table 1:** Sample characteristics.

Interview	Gender	Age	Profession	School	Region	Deals with transcultural situations?
1	M	34	Mathematics teacher	Middle school	Paris	Yes
2	F	35	Head guidance counselor	Middle school	Paris	Yes
3	F	52	Spanish teacher	Middle school	Paris	Yes
4	F	55	French teacher	Middle school	Paris	Yes
5	F	53	English teacher	Middle school	Paris	Yes
6	F	38	School nurse	Middle school	Paris	Yes
7	F	45	Director	Schooling association	Paris	Yes
8	F	42	Teacher	Schooling association	Paris	Yes
9	F	58	Teacher	Schooling association	Paris	Yes
10	M	50	History teacher	Middle school	Paris	Yes
11	F	52	School nurse	Middle school	Paris	Yes
12	F	55	School doctor	Middle school	Paris	Yes
13	F	40	Mathematics teacher	High school	Bourgogne Franche-Comté	Yes
14	F	40	English teacher	High school	Bourgogne Franche-Comté	Yes
15	F	29	History teacher	High school	Bourgogne Franche-Comté	Yes
16	M	50	Head guidance counselor	High school	Bourgogne Franche-Comté	Yes
17	F	60	Assistant principal	High school	Bourgogne Franche-Comté	Yes
18	F	51	School nurse	High school	Bourgogne Franche-Comté	Yes
19	M	55	Principal	High school	Bourgogne Franche-Comté	Yes
20	F	50	English teacher	High school	Bourgogne Franche-Comté	Yes
21	F	45	French teacher	High school	Bourgogne Franche-Comté	Yes
22	F	35	English teacher	High school	Bourgogne Franche-Comté	Yes
23	F	60	School doctor	Local education authority	Bourgogne Franche-Comté	Yes
24	F	53	French teacher	Middle school	Bourgogne Franche-Comté	Yes
25	F	30	Educational assistant	Middle school	Bourgogne Franche-Comté	Yes
26	M	47	Principal	Middle school	Bourgogne Franche-Comté	Yes
27	F	50	Biology teacher	Middle school	Bourgogne Franche-Comté	Yes
28	M	50	School nurse	Middle school	Bourgogne Franche-Comté	Yes
29	F	59	French teacher	Middle school	Bourgogne Franche-Comté	Yes
30	F	36	French teacher	Middle school	Bourgogne Franche-Comté	Yes

### Procedure

All participants were interviewed by one of two researchers (RM and LB). All in-depth interviews were tape-recorded, transcribed, and anonymized. Pseudonyms are used when necessary. All participants provided oral informed consent before their inclusion in the study and were asked to repeat it at the beginning of the recording. Because the ethics committee considered that written consent might weaken the anonymization process by linking names to the consent, it required audio-recorded oral consent. An interview guide for in-depth interviews was developed and included open-ended questions that focused on how participants understand school refusal and the practices that might shape the health care pathway of youths from families with transcultural backgrounds. The in-depth interviews sought to obtain a detailed, rich understanding of the topic of interest. The participants' experiences, behaviors, feelings, and attitudes were probed deeply to identify underlying concepts that the researchers analyzed to generate a theory that provided a deeper understanding of the research topic.

In-depth interviews are more structured than narrative interviews as the topic discussed is directed by the researcher, and they rarely involve stories or life histories. They do, however, allow the participant to communicate much more freely and to provide more detailed descriptions than in semistructured interviews. The precise details of the research questions were not revealed during the interviews, to prevent them from influencing the material obtained or “leading” the participants to particular responses. Rather, the general area of interest was explained to the participants, and the interviewer directed further conversation based on the responses. In accordance with GT's inductive methodology, the initial open questions used in the interview guide were based on the international literature, but kept evolving over the course of interviews, as they were analyzed. The interview guide included questions such as: “Can you tell me about the situations of school refusal you've encountered?”, “What does ‘school refusal' mean to you?” and “How do you manage these situations?”

As required by GT, the data analysis, sampling of new participants by in-depth interviews, and theoretical development continued simultaneously until saturation was reached and a new theory was constructed about our topic (28). LB and RM independently coded all interviews. During the analysis, categories were created by gradual coding of the data, which were constantly compared with those from new interviews, with the codes modified as needed. Triangulation of the analysis, which guarantees the quality of individual coding, took place during monthly meetings of our research group (LB, RM, JB, and MM).

## Results

**Figure 1:**
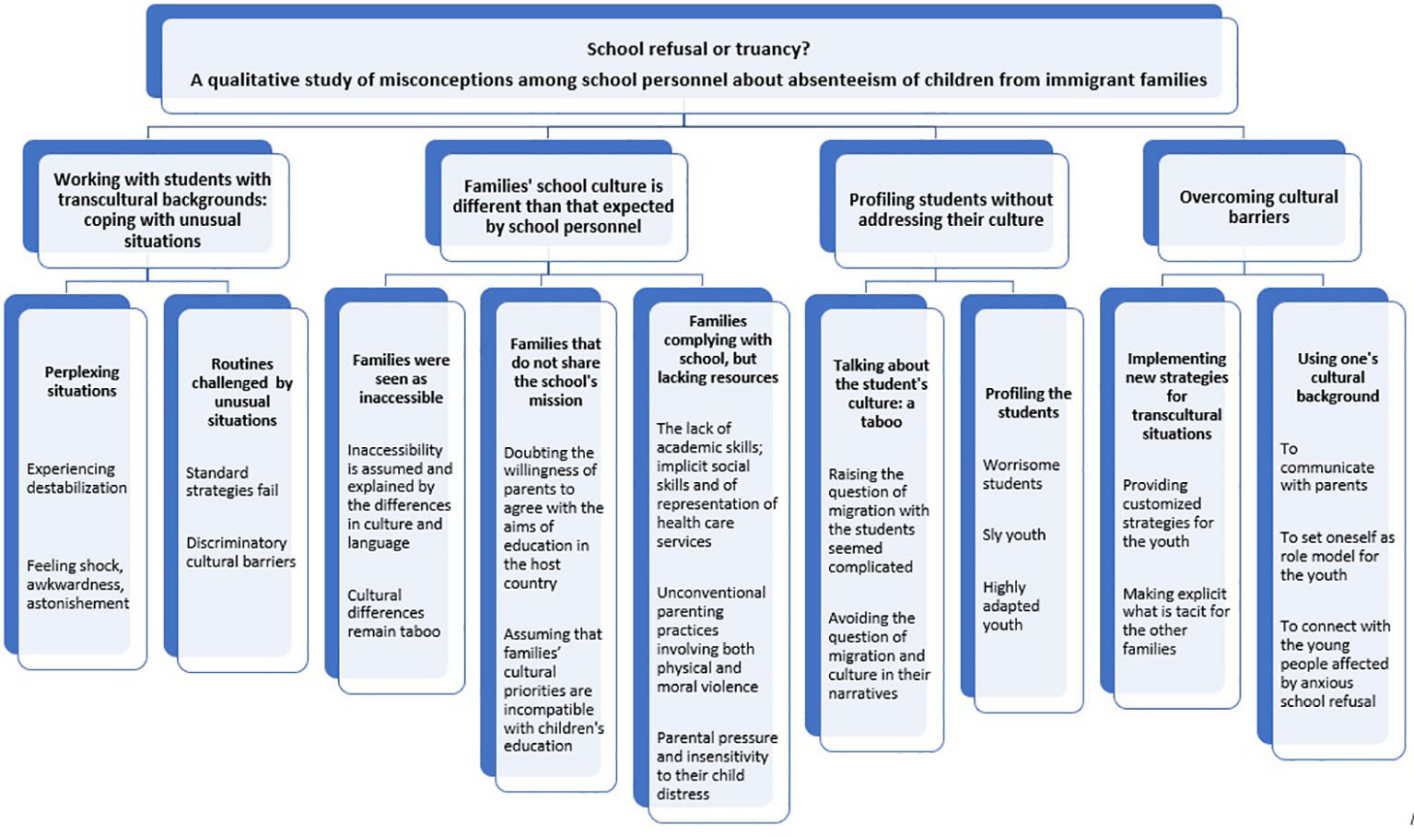
Flow chart showing the interview topic (level 1), themes (level 2), subthemes (level 3), and higher-level codes (level 4).

The practices of school personnel for dealing with school refusal among children with transcultural backgrounds were captured in four main themes: (1) working with students with transcultural backgrounds: coping with unusual situations, (2) families' school culture is different than that expected by school personnel, (3) profiling students without addressing their culture, and (4) overcoming cultural barriers (see **Figure 1**). These practices also underlined the differences between the immigrant populations in the Paris area and in the Bourgogne-Franche-Comté region. In Paris, the situations mentioned most often concerned second-generation immigrants from North and sub-Saharan Africa and from Asia, while in Bourgogne, encounters with travelers (largely various groups of Roma, also administratively designated as “*gens de voyage*” in France) and young political refugees from the Middle East were more frequent. The participants reported no situations involving school refusal among the young refugees. The main results are shown in **Figure 1**, and the transcripts are summarized in **Table 2**.

**Table 2:** Illustrative quotations from transcripts.

Themes	Categories	Quotes
**Working with students with transcultural backgrounds: coping with unusual situations**		
**Perplexing** **situations**	Experiencing destabilization	(Talking about Chinese sisters): “they are severely um … affected … by something that is, in my opinion, really hard to deal with. For them. *Because I've rarely seen that*, uh…” (French teacher, 4) “There's a student who's from Kosovo, arrived 5 years ago, [and] who I had last year in Year 10, she was absent a lot for, apparently for depression, so I didn't know more about than that, it's hard to say that she was one of the students, that's all, *it was really something a little different*.” (French teacher, 27)
	Feeling shock, awkwardness, astonishment	(About a young sub-Saharan girl): “You have this girl, who is basically apathetic. You'll see who can be discomfited! The ‘I don't care' type. When I say impassive, *it's impressive*. You have the impression that nothing is happening, really, it's the right term.” (School nurse, 6) (Talking about a Kabyle youth): “I had a young man who was failing. He wasn't coming [to school]. We were very *shocked*. When the parents were able to say, it's the mother who explained, that ‘we can't help him with school'. The mother was illiterate.” (English teacher, 5)
**Routines challenged by unusual situations**	Standard strategies fail	“It complicates things. And it wasn't only not speaking the language. When you don't understand, yourself, well you don't know, it's hard to say ‘You have to be careful about this.' If the person understands one word out of two, the whole meaning might be distorted. And then, the understanding of the French educational system, its requirements. Finally everything is more complicated, at that point it's much harder to work, it's harder to understand the causes, and as a result, harder to draw conclusions.” (Math Teacher, 1)
	Discriminatory cultural barriers	“There's a parent, I believed he wouldn't speak very well. In fact, he speaks very very well.” (School nurse, 6) “Parents don't express themselves very well in French, so the children are sort of left to themselves. They're in school, but it becomes complicated.” (School nurse, 6) “We've forgotten to say, ‘Why? Why is he not managing to meet our requirements?' For us, it was ‘immature' or ‘he doesn't listen!' And, well, no, there is nonetheless a minority, a numerous minority who don't fit into our explanations.” (English teacher, 5)
**Families' school culture is different than that expected by school personnel**		
**Families were seen as inaccessible**	Inaccessibility is assumed and explained by the differences in culture and language	“There are cultures—without necessarily being misogynist—*it's cultural …* People who might be bothered, there, if it were a guy, sometimes, I'm sure that, I think that it could change the situation.” (School nurse, 6)
	Cultural differences remain taboo	“It's hard, [the] single-parent families, it's complicated, because we also have cases of polygamy, anyway, not enormously but um, it's not known *it's not said, it's not written*, but we know … *Because we asked a question, and we didn't understand*, and we know that it's polygamy. Single-parent, that doesn't say much.” (School doctor, 13)
**Families that do not share the school's mission**	Doubting the willingness of parents to agree with the aims of education	“Among the students who I knew they were victims of anxious school refusal, there are sometimes families that are extremely caring, concerned and put time into their children's education, and others sometimes can be a little less so, uh” (History-geography teacher, 11)
	Assuming that families' cultural priorities are incompatible with children's education	“There is, sometimes, a lack of interest in school that can be linked to the family's culture. A child, those they call the travelers, there are a lot of them around here. And the school culture is truly under … underestimated, undervalued. The idea is to be able to work as soon as possible, manual labor, and soon.” (Principal, 41) “The travelers, it depends on the season. There's a semi-chronic absenteeism, in these families. At harvest time, the kids disappeared, they reappeared after. For me, *it's cultural*. The lifestyle is not compatible with on-going education.” (French teacher, 38) “When you come from a country where school is more a question of luck and an optional right, ok, he goes to school occasionally and there is not so much regular follow-up of lessons. There are some parents who also don't understand, the necessity that the child be there” (Math teacher, 1) “I see families of sub-Saharan origins especially, school isn't more or less important than anything else. They don't see the stakes of school in our civilization, clearly.” (School doctor, 13) “Religion is starting to take a large role in our society. This religion makes people … stand out by the fact of belonging to a group. And so school has no more reason to exist. The older generation, where, on the contrary, the children were super-proud of succeeding, for their parents, and the parents were very proud of their children's success, and of their total integration in the country. You were supposed to be completely integrated, not show your difference. But now, the aim is to show your difference.” (Spanish teacher, 3)
**Families complying with school, but lacking resources**	The lack of academic skills	“The families don't all have, necessarily, *the resources* that would enable them to ensure, “success” (in quotation marks), but can nonetheless have an extremely negative view of failure.” (History and geography teacher, 11)
	The lack of implicit social skills	“He started to not come anymore … But really, fear in the belly, you know! We had to telephone them to find out what was going on. They came. The mother explained.” (English teacher, 5)
	The lack of representation of health care services	“Sometimes *we have trouble making the parents come in*, it's not in their culture, psychiatrists … well there are plenty of civilizations where it's not in the culture … to make them understand that there is care that is necessary.” (School doctor, 13) “Culturally, sometimes, with the Asian population, we have trouble getting them to adhere to care.” (School nurse, 12)
	Unconventional parenting practices involving both physical and moral violence.	“It is striking to observe families from foreign countries, who seem, in relation to school, to be conscious of the importance of the institution, but whose response is a sort of a condemnation that is demonstrated like that, publicly, and that can even take sometimes violent forms.” (History-geography teacher, 11) “It's the parents who had the most trouble expressing themselves in French [who] were the most severe with their children. I had asked them to come in to see me, because his grades weren't good, and then he got slapped in the head. It was a question of honor. The parents feel very guilty each time that there's a report of dropping out. You see it in their attitude, even though it's expressed differently.” (Biology teacher, 42)
	Parental pressure and insensitivity to their child distress	“The parents demanded that it continue to work well. He was coming less and less often to class, and the family demanded that he be at school.” (School nurse, 12) “Chinese … it's silence, they say nothing, [the parents] say nothing, everything is fine … Clams … but no, it'll be ok, they clench their teeth, and then they come.” (School doctor, 13) “Parental pressure is a factor in school phobia. There's greater parental pressure in the Asian community than in the other communities.” (School doctor, 13)
**Profiling students without addressing their culture**		
**Talking about the students' culture: a taboo**	Teachers' difficulty to refer to culture and migration in their narratives	“I asked one of them where she had been in school, because I didn't dare ask if she was born in France.” (French teacher, 4)
	Using euphemisms and conniving allusions	“You rarely have, uh…'René', most of the time you have ‘Mamadou.' Ok, you see?” (English teacher, 5)
**Profiling the students**	Worrisome students	(Talking about two Chinese sisters): “They do everything they can to be forgotten and they succeed. They don't move. They don't gesture. They don't catch my eyes. I asked one of them to tell me if, because they are … I said to myself: there, maybe they don't speak French well, they are completely lost.” (French teacher, 4)
	Sly youth	“We have a lot of first-generation immigrants who do not speak French at all. And as a result, no matter how many letters you send them, no matter how many times you call, sometimes you get the student, you don't know, sometimes he fakes it! He picks up and says, “Yes yes I'll tell him.” (Math teacher, 1)
	Highly adapted youth	“We have a lot of foreign students. They invest enormously in school because they understand that it is their only path to salvation. These children [with anxious school refusal] are vulnerable, from an emotional point of view, a little overprotected. Those [immigrant youth], inversely, they are torn from their parents, torn from their family, torn from their friends, and they are super happy to be here.” (Head Guidance Counselor, 19) “They have a power of adaptation, finally … there are two kids who came from Italy, and who came perhaps from Syria before. And so they learned, they already speak French really well, it's incredible! (Educational assistant, 39)
**Overcoming cultural barriers**		
**Implementing new strategies for transcultural situations**	Providing customized strategies for the youth	“Sometimes we let the students leave, because they have a psychiatry appointment and their parents mustn't know. Because that can put them in danger.” (Head Guidance Counselor, 2)
	Making explicit what is tacit for the other families	“It can be hard to make [Chinese parents] understand that there is treatment that is necessary, so that they want to hear uh … it's complicated for their child … that their child, he's not well.” (School doctor, 13).
**Using one's cultural background**	To communicate with parents	“I come from another culture too, my family is Iranian. So, I know how to talk to parents who believe that all you have to do is say, ‘listen to your teacher, listen to your teacher.' Because there's no agreement about values, especially in middle school.” (English teacher, 5)
	To set themselves as role model for the youth.	“I'm originally Algerian and there've been students from North Africa and who weren't succeeding. [They said] ‘in any case, I'm stupid, my parents can't read'. So I explained to them, well, my parents couldn't read or write either, but I passed the agreg [advanced civil service test]. I think that also affected them. Anything is possible, and then as a result I set up personalized help for them. [They told me] ‘you give us personalized help, finally someone who listens to us, who considers us.'” (English teacher, 29)
	To connect with the young people affected by anxious school refusal	“There remains, even for these youth, a desire for ‘cultural nourishment.' History and geography are often very important for these youth who have … anxious school refusal … because they always find an association with their past, their culture, their roots.” (Director, 7)

### Working With Students With Transcultural Backgrounds: Coping With Unusual Situations

#### Perplexing Situations

The participants reported feeling destabilized by their encounters with otherness. Many of them described the psychological distress of these students from transcultural background as perplexing, difficult to understand, and mysterious.

“(Talking about Chinese sisters): they are severely um … affected … by something that is, in my opinion, really hard to deal with. For them. Because I've rarely seen that, uh…”

(French teacher, 4).

“There's a student who's from Kosovo, arrived 5 years ago, [and] who I had last year in Year 10, she was absent a lot for, apparently for depression, so I didn't know more about it than that, it's hard to say that she was one of the students, that's all, it was really something a little different”

(French teacher, 21).

Participants described feeling shocked, awkward, and astonished. Accordingly, several of the participants used the term type when they were asked to describe unexpected behaviors of youths from transcultural background.

“(About a young sub-Saharan girl): You have this girl, who is basically apathetic. You'll see who can be discomfited! The “I don't care” type. When I say impassive, it's impressive. You have the impression that nothing is happening, really, it's the right term”

(School nurse, 6).

#### Routines Challenged by Unusual Situations

These unusual situations jeopardized these school personnel's regular practices by defeating their standard strategies. They rattled their pedagogic reasoning, upsetting everything from their communication to their ability to find practical solutions. A math teacher (participant n°1) noted

“It complicates things. And it wasn't only not speaking the language. When you don't understand yourself, well you don't know, it's hard to say “You have to be careful about this.” If the person understands one word out of two, the whole meaning might be distorted. And then, the understanding of the French educational system, its requirements. Finally everything is more complicated, at that point it's much harder to work, it's harder to understand the causes, and as a result, harder to draw conclusions”.

Obstacles are often embodied by language barriers. In this sense, a language barrier was assumed before the first encounter with the parents. This barrier, when it exists, is often considered equivalent to parental inability to raise and educate their children responsibly. Only one of the 30 participants, however, pointed out that prejudices shape educators' practices regarding numerous children who do not conform to the way they usually understand learning difficulties. No other participants acknowledged any bias. To illustrate these discriminatory cultural barriers, the teacher described a Kabyle youth whom the school personnel had considered a “truant” until a conference with the family showed that the behavior was in reality school refusal. This dialogue occurred only because the school personnel took the initiative to call the parents. The parents had not dared to contact the school and appeared to accept the child's absences; their apparent failure to secure their child's attendance at school led to the conclusion the child's behavior could not be school refusal but was instead truancy. This shows the potential for misdiagnosis between truancy and school refusal, according to Berg's criteria, when applied to children of migrant families.

“I had a youth who was failing, he was afraid to come [to school] because he knew he hadn't done his homework, that we were going to reproach him. We had created a very onerous climate. At a certain point, he started to not come anymore. But really, he was petrified, you know! We were very shocked that … when the parents were able to say … because the youth, he had never said. We had to telephone them to find out what was going on. They came, and the mother explained that “we can't help him with school work”. The father works from morning til night, and the mother was barely literate. So he had fallen behind. We, by our demands—which were justified—we had just forgotten to ask ourselves why he wasn't managing to meet our requirements. For us, he was “immature”, ‘he doesn't listen”. And, well, no, there is nonetheless a minority, a numerous minority, who don't fit into our explanations. This kid was petrified to come to school. It can be truly overwhelming”

(English teacher, 5).

### Families’ School Culture Is Different Than That Expected by School Personnel

#### Families Were Seen as Inaccessible

Both school medical personnel and teachers expressed their impression that immigrant families are inaccessible. A language barrier was assumed before the first talk. Beyond the language issue, participants explained this inaccessibility by reasons such as differences in cultural representations. For example, a school nurse (participant n°6) saw the difference in male-female relationships as accounting for her unease when talking to migrant fathers.

“There are culture—without necessarily being misogynist—it's cultural … People who might be bothered, there, if it were a guy, sometimes, I'm sure that, I think that it could change the situation.”

However, talking openly about cultural difference remained taboo for the participants. Polygamy, for instance, is a cultural practice common in several African countries, but forbidden in France. Thus, when the participants understood that the students' parents were living in this illegal form of marriage, they evoked the topic only indirectly, through allusions. Similarly, participants who had contact with immigrant families described diverse school-related representations, behaviors, and skills indirectly. Moreover, they understood the difficulties they encountered in dealing with these families in two different ways:

#### Families That Do Not Share the School's Mission

Sometimes, participants doubted the willingness of some immigrant parents to agree with the aims of education in the host country. They questioned whether some parents intended to play their customary role in French society, where parents are expected to help their children with their school work and thus support and promote their academic achievement. Some participants implied that some parents might be inattentive to their child.

“Among the students who I knew they were victims of anxious school refusal, there are sometimes families that are extremely caring, concerned, and put time into their children's education, and others sometimes can be a little less so, um…”

(History-geography teacher, 10).

Other parents were seen as uninterested in the child's schooling. Participants explained this by both cultural reasons and priorities incompatible with the children's education and integration into the education system. Some personnel considered that parents were not prepared for the importance of school in French society:

“When you come from a country where school is more a question of luck and an optional right, ok, he goes to school occasionally and there is not so much regular follow-up of lessons. There are some parents who also don't understand, the necessity that the child be there”

(Math teacher, 1).

According to some participants, some families, including immigrants, have shifted their life goal from school success to religion and religious membership. The older generations of migrants had sought assimilation through school achievement and the concealment of any cultural difference. However, the younger generation appeared to claim their pride in aspects of their identity setting them apart from the culture of the host country, such as religion. As one teacher said,

“Religion is starting to take a large role in our society. This religion makes people … stand out by the fact of belonging to a group. And so school has no more reason to exist. The older generation, where, on the contrary, the children were super-proud of succeeding, for their parents, and the parents were very proud of their children's success, and of their total integration in the country. You were supposed to be completely integrated, not show your difference. But now, the aim is to show your difference”

(Spanish teacher, 3).

#### Families Complying With School, but Lacking Resources

Immigrant parents were sometimes depicted as, at most, willing to help their child presenting school refusal by encouraging them to attend. School personnel described parents who, despite their goodwill, lacked academic and social skills and had inaccurate representations of mental health care and inappropriate educational attitudes that could account for their children's school issues. Some families who agreed with the school culture in France and its aims (academic achievement) lacked the academic skills necessary to help their child:

“The families don't all have, necessarily, the resources that would enable them to ensure, “in quotation marks” success, but can nonetheless have an extremely negative view of failure”

(History and geography teacher, 10).

Participants stressed parents' lack of social skills as an explanation for some immigrant parents' relationships to institutions, such as school and health care services. For instance, some parents did not grasp that they were expected to be the ones to call school personnel and ask for a meeting. The lack of this implicit social skill required to interact with the school sometimes led to misunderstandings. School medical personnel also attributed the difficulty in referring immigrant families to mental health care services to a lack of representations of integrated youth health care services (29, 30).

“Sometimes we have trouble making the parents come in, it's not in their culture, psychiatrists … well there are plenty of civilizations where it's not in the culture … to make them understand that there is care that is necessary”

(School doctor, 12).

“Culturally, sometimes, with the Asian population, we have trouble getting them to adhere to care”

(School nurse, 11).

Participants sometimes mentioned parents' inappropriate or unusual attitudes towards their children with emotional or academic difficulties. During parent–teacher meetings at school, participants noticed unconventional parenting practices involving both physical and moral violence. Parents sometimes considered public physical violence to be a way to demonstrate their efforts to secure school attendance, despite their inability to provide the child with effective support.

“It's striking to observe families from foreign countries, who seem, in relation to school, to be conscious of the importance of the institution, but whose response is a sort of a condemnation that is demonstrated like that, publicly, and that can even take sometimes violent forms”

(History-geography teacher, 10).

Some participants underlined the moral dimension of the relationship of these families with the school, reflected in their willingness to show their honor, responsibility, and respect for school rules:

“It's the parents who had the most trouble expressing themselves in French [who] were the most severe with their children. I had asked them to come in to see me, because his grades weren't good, and then he got slapped in the head. It was a question of honor. The parents feel very guilty each time that there's a report of truancy. You see it in their attitude, even though it's expressed differently”

(Biology teacher, 27).

Physical violence was not the only inappropriate means by which immigrant parents expressed their efforts to get their child to school. Parental demands related to school attendance can also be viewed as a kind of insensitivity to their child's distress and as rigidity in their relationship with school personnel, as these statements by school medical personnel show about some families from China.

“The parents demanded that it continue to work well. He was coming less and less often to class, and the family demanded that he be at school”

(School nurse, 11).

“Chinese … it's silence, they say nothing, [the parents] say nothing, everything is fine … Clams … but no, it'll be ok, they clench their teeth, and then they come”

(School doctor, 12).

Some participants feel that this “unsympathetic” support from some immigrant parents for the schooling of their child is parental pressure that can lead the child to develop school refusal:

“Parental pressure is a factor in school refusal. There's greater parental pressure in the Asian community than in the other communities”

(School doctor, 12).

To conclude, these two different lines of thinking—intentional lack of parental efforts, on the one hand, and lack of resources, on the other—were often observed in interviews of the same participant. This reflects their constant ambivalence and doubts when trying to decide whether to assign responsibility (internal causality in the family) or point out determinism (external causality) in each situation.

### Profiling Students Without Addressing Their Culture

#### Talking About the Students' Culture: A Taboo

Participants appear to find raising questions with their students about the students' difference, foreignness, to be sensitive, potentially offensive, just as they did in talking with and about the families. They hesitated to talk about culture with the students, speaking carefully and sometimes using alternate euphemisms, such as geographic origin, or ethnic group, migration status, or other categories.

“I asked one of them where she had been in school, because I didn't dare ask if she was born in France.”

(French teacher, 4).

They also found it difficult to refer to culture and migration in their narratives; this was true from the perspectives of both style and content. To name the otherness of these young people, school personnel used several different qualities and mixed them, for example, switching between ethnic dimension and migration status. Several levels of language were used, varying from formal words (such as “first-generation immigrants”) to more colloquial ones (“Chinese”). Sometimes participants even tried to overcome their unease by inviting the interviewer to share conniving allusions to foreign names, to underline their exoticism, their difference.

“You rarely have, uh…”René,” most of the time you have “Mamadou.” Ok, you see?”

(English teacher, 5).

#### Profiling Students

Despite their difficulties dealing with the topic of the students' origins, some participants drew collective portraits of the immigrant youth, with descriptions very different in Paris and Bourgogne. Some profiles were idealized, others more pejorative.

In Paris, interviews were conducted in multicultural and multiethnic schools, with first- and second-generation immigrant students. Some participants reported worrisome students who made efforts to become invisible to their classmates and to the teacher. Paradoxically, this attitude apparently helped to call them to the attention of the teacher, who was impressed by this effort at invisibility. Although non-immigrant youth with school refusal also worked at hiding themselves in plain sight, at least some participants seemed to explain this attitude as transcultural, through the language barrier, for instance:

“(Talking about two Chinese sisters): They do everything they can to be forgotten and they succeed. They don't move. They don't gesture. They don't catch my eyes. I asked one of them to tell me if, because they are … I said to myself: there, maybe they don't speak French well, they are completely lost”

(French teacher, 4).

Some immigrant youths were perceived as sly, trying to take advantage of their parent's cultural distance from the school, making use of the parent's lack of knowledge of some social codes and practices to reap benefits or avoid penalties in some situations. The language barrier between their parents and school personnel was sometimes used similarly:

“We have a lot of first-generation immigrants who do not speak French at all. And as a result, no matter how many letters you send them, no matter how many times you call, sometimes you get the student, you don't know, sometimes he fakes it! He picks up and says, “Yes, yes I'll tell him”

(Math teacher, 1).

In Bourgogne, young refugees were mostly described as highly adapted youth, strongly invested in their schooling and expressing no difficulties. The head guidance counselor explained this excellent involvement by the school's role as their only lifeline. This picture of the resilient young refugees showing interest in school despite adverse life events was opposed to a portrait of youths with school refusal who were born in France of French parents.

“We have a lot of foreign students. They invest enormously in school because they understand that it is their only path to salvation. These children [with anxious school refusal] are vulnerable, from an emotional point of view, a little overprotected. Those [immigrant youth], inversely, they are torn from their parents, torn from their family, torn from their friends, and they are super happy to be here”

(Head Guidance Counselor, 16).

Some personnel underlined these students' ability to adapt to the French educational system and the French language.

“They have a power of adaptation, finally … there are two kids who came from Italy, and who came perhaps from Syria before. And so they learned, they already speak French really well, it's incredible!”

(Educational assistant, 25).

### Overcoming Cultural Barriers

#### Implementing New Strategies for Transcultural Situations

The perturbing transcultural situations encountered by the participants obliged them to adopt a reflexive attitude and to carefully think through the circumstances to adapt their practices. Several developed customized strategies for these youths, sometimes collectively organized with the school team, sometimes more individually. One example was the provision of confidential support to students, unbeknown to their relatives. As the head guidance counselor (participant n°2) pointed out:

“Sometimes we let the students leave, because they have a psychiatry appointment and their parents mustn't know. Because that can put them in danger.”

With immigrant families, personnel may have to state explicitly that which is understood tacitly by other families. This pedagogy can be done either through official institutional activities or in a less formalized manner, through direct conversation.

#### Using One's Cultural Background

Among the most creative ways participants supported families, one involved the use of their own cultural background to communicate with parents and to undertake a sort of decoding of the values espoused by the school system of the host country.

“I come from another culture too, my family is Iranian. So, I know how to talk to parents who believe that all you have to do is say, “listen to your teacher, listen to your teacher”. Because there's no agreement about values, especially in middle school”

(English teacher, 5).

These participants used their cultural background and their academic achievement to set themselves as role models for the youth. They also offered them personalized review courses:

“I'm originally Algerian and there've been students from North Africa and who weren't succeeding. [They said] “in any case, I'm stupid, my parents can't read.” So I explained to them, well, my parents couldn't read or write either, but I passed the agreg [advanced civil service test]. I think that also affected them. Anything is possible, and then as a result I set up personalized help for them. [They told me] “you give us personalized help, finally someone who listens to us, who considers us”

(English teacher, 22).

Subjects, such as history or geography, linked to the culture are another means of connecting with the young people affected by school refusal. As one school director (participant n°7) said:

“There remains, even for these youth, a desire for “cultural nourishment”. History and geography are often very important for these youth who have … anxious school refusal … because they always find an association with their past, their culture, their roots.”

## Discussion

### In Which Cases of School Refusal Is the Issue of Culture Noticed, and in What Ways?

Only 30 school personnel interviewed about school refusal reported working with students with transcultural backgrounds. Their interviews underlined the discomfort that they experienced when they had to deal with families with cultural differences. The French school system is steeped in the ideology of the *République laïque* and antiracism, inherited from the Enlightenment philosophers who proclaimed their “indifference to differences” on the public scene, that is, to say, in institutions such as hospitals and schools. These imposed neutral priorities sometimes lead school personnel to avoid dialogue about cultural and ethnicity issues when they deal with minorities. This avoidance produces the taboos described in the *Results* section and therefore requires specific attention in “color-blind” societies that are in reality multicultural. Treating differences as taboos may force school personnel to use superficial and thus biased concepts of culture in their dealings with immigrant families.

The participants' narratives when speaking of SAPs in transcultural contexts revealed that their encounter with otherness and cultural difference was unexpected. These transcultural encounters left them uneasy, troubled, even stunned, emotions transmitted by the words and tone of their responses. To some extent, this recalls some traits of posttraumatic narratives. These unusual situations dissolved their professional self-confidence, leaving them passive and helpless in the face of this experience. The puzzlement expressed in their answers on the topic of school refusal by youths from transcultural backgrounds might thus mirror the traumatic experience of their encounter with otherness, perhaps recalling the concept of vicarious traumatization (31). Thus, the non-standard practices subsequently developed by some participants for dealing with these situations might be seen as defensive strategies to enable them to again become an active participant, thus recovering their professional identity. Inversely, when students belonging to the dominant culture exhibited SAPs, school personnel never mentioned cultural factors because, in that situation, they perceive no reason to think about or question the implicit cultural beliefs and values that they share with the family.

### The School Personnel's Ambivalence Toward Cultural Factors

Participants' responses to school absenteeism of children from transcultural backgrounds vary, but seem to share a common denominator: the necessity for school personnel to shift from prereflexive adjustment to a situation to conscious reflexivity. Working with immigrant families often leads social workers and care providers to discover previously unseen modes of socialization, unique for each family, as underlined by Lahire (32). Lahire argues that the greater the difference between how the family and the teacher understand school requirements, the less likely the student is to achieve success in school (33). This risk of failure is even greater if, as is most often the case, school personnel are unaware of the families' internal approach or reasoning in relation to school. One example of this, illustrated by the participants' descriptions of their experiences, is the issue of corporal punishment at school. Physical punishment was previously authorized by law in French schools, but is now illegal. French parents know today that this written law prevails over customary laws that did to a certain extent allow violence against children. This may explain why participants were shocked by the parental corporal punishment in front of them, behavior from another time regarded as retrograde. Thus, participants may not have even considered the possibility that these migrant families have a different attitude or approach to corporal punishment, one sustained by different sociological and cultural processes, as Delanoë has pointed out (34).

The encounter with families with transcultural backgrounds confronts school personnel with a different cultural approach to school (or care) from their own. The personnel lack both theoretical training in transcultural issues and practical experience with migrant families. The unusual situations they faced defied their usual work strategies, which are appropriate for families in intracultural contexts. Thus, school personnel's understanding of the behavior of both students and parents can be riddled with prejudices and stereotypes. There is accordingly a risk of negative bias in how school personnel perceive immigrant families: they often meet the student or his or her family for official appointments or when the family is summoned for disciplinary issues. These negative experiences increase the risk that personnel will form negative, often inaccurate impressions of these immigrant families, for example, by perceiving some parents as abdicating their parental responsibility by their manifestation of a different relationship to the school (35). Such misunderstandings in turn may well influence the staff's current and future representations, perhaps resulting in a “self-fulfilling prophecy” (specifically, a “golem” effect), with potential prejudicial outcomes: on one hand, reinforcement of the negative representations about the immigrant parents and thus less likelihood of working positively with them, because they are discredited in advance and so easily pictured as “abdicating” (36); on the other hand, students' subjective disengagement from their relations with the school, as a strategy to preserve their self-image and to avoid confirming a negative stereotype of their cultural group (37).

#### Cultural Countertransference Leads to Misdiagnosing School Refusal as Truancy

Devereux theorized cultural countertransference as the sum total of an observer's implicit and explicit reactions, conscious and unconscious, to the observed object in a transcultural situation (38). For the purposes of this study, this includes all the conscious and unconscious prejudices and representations, both negative and positive, that school personnel of a given culture will experience when working with a youth or a family from another culture. These representations are organized around the tension between exclusion on the one hand, and the validation or even idealization of cultural difference, on the other. An example is how immigrant parents are sometimes treated as unfit by some personnel, who at the same time want to protect the children from their parents' supposed neglect. The cultural countertransference thus influences the perception as well as the reasoning of the participant—both underpinned by the affective polarization between rejection and validation.

School refusal is an interesting disorder for exploring the impact of cultural countertransference. When immigrant parents had not dared to contact the school personnel, their apparent failure to secure their child's attendance at school led the participants to the conclusion the student's behavior could only be defined as truancy. Thus, school refusal may well be misdiagnosed as truancy, according to Berg's criteria, when applied to children of migrant families. In this sense, the participants of this study understood their migrant students' absenteeism as truancy instead of school refusal. Several lines of reasoning thus appear in these narratives about immigrant families and may yield various practical consequences. School personnel attribute intentionality to the immigrant families' positioning in relation to the school and consequently also ascribe to them responsibility for the school refusal. At the same time, however, they perceive a possible cultural determinism, which they may interpret as a social handicap, explained by failure to master the school's norms of conduct, because some families lack the explicit and implicit knowledge, attitudes, and skills required to communicate and interact with school personnel in a way the latter consider normal. For youths with transcultural backgrounds, the participants interpreted school absenteeism as a result of behavioral or mood problems, the family's lack of interest in school, language problems or poor grades. The sole exception was the English teacher of Iranian descent (participant n°5), who went to the trouble to call the family of a truant-like student and thus discovered that the youth was “petrified” by fear and presented with school refusal. The tension between these two opposing narratives thus reflects the complexity of the participants' work with immigrant families and their constant ambivalence.

School personnel's experiences with the unconventional behavior of families that do not share their school culture lead them to interpret it as deviance, whether intentional or determined. These opposing tensions are found too in their diagnosis of situations. From one perspective, they diagnose school refusal, accepting the idea of external determinants that overwhelm the youth and his or her family and thus a certain determinism. From another perspective, they consider the youth a school truant, a label that presupposes that he or she made a choice or is otherwise responsible for the situation. Thus, students with transcultural backgrounds can challenge the agreed-upon understanding of school refusal as taught to education professionals. The final diagnosis will of course depend very much on other factors along any given youth's pathway from school onward, including perhaps to a hospital. These include especially the mutual influence of the teachers, counselors, psychologists, doctors, and family members beside them. Finally, the cultural differences in these examples appear to be seen most often as a barrier to the school personnel's work, although they do not always block this work. Thus, participants, reporting being destabilized in their professional identities by their encounter with otherness, might act out their cultural countertransference aggressively, for example by promoting referral to court or social services, and simultaneously rationalize this decision.

#### Addressing Diversity to Help Immigrant Youth With School Refusal

Different subgroups in our research group focused on the viewpoints of school personnel, migrant parents (39), child psychiatrists (40) and adolescents (41), and their attitudes about school refusal in transcultural contexts. The results of this study of school personnel and their difficulties with children presenting with school refusal in transcultural situations, mirror the findings of the groups studying parents, psychiatrists, and adolescents. As Rosenthal et al. stressed, the immigrant parents of children presenting with school refusal may have misrepresentations of the school and, of their child's difficulties and may also lack the cultural codes needed to interact effectively with school personnel or doctors.

Most of the transcultural contexts described by the participants expressed a negative vision of the students' culture, which was seen as an obstacle in their relationship to education. Payet (42) described the issue in dealing with ethnicity at school in France as a balance between overdetermination and invisibilization of otherness. On the public scene, strangeness cannot be openly talked about, whereas school personnel can talk about it “backstage”. Moreover, the tension between this ideology and the work these personnel do in a daily life that is increasingly ethnicized risks promoting a hesitation between affirmation and stigmatization when talking about cultural differences (43). In color-blind societies, the tension between recognizing and ignoring questions of culture in schools might compromise the school achievement of young immigrants (44–46). Mansouri, for example, linked the school difficulties of immigrant youth whose parents were born in former French colonies (North Africa and sub-Saharan Africa) to a postcolonial dimension (47). Thus, two conditions might lead school personnel to develop discriminatory reasoning and youth to perceive discriminatory attitudes: 1) the unconscious symbolic violence (of the school personnel against the youth) inherited from the colonial past and reproduced in the “here and now” of the present relationship with the pupils; and 2) the youth's unconscious knowledge of this past, which he or she has received through transgenerational transmission. The youngster's subsequent feeling that the egalitarian ideal has been betrayed could create a dominant/dominated relationship mirroring the colonizer/colonized (or master/slave) relationship (48).

However, some participants creatively implemented new ways to deal with these situations, by using the cultural difference for mediation. They thereby demonstrated that the outcome of working with immigrant families is not a foregone (negative) conclusion. Many participants experienced problems with students with transcultural backgrounds, including those displaying SAPs, differently than they did their usual work and developed new strategies to deal with it. Exploring this dimension in the standardized assessment scales exploring school environment, such as ISAP (49), might enhance their clinical efficacy, by giving a clearer picture of each situation, and especially of the interactions between the youth and the school personnel. Including a transcultural dimension in early intervention programs, such as for psychosis (50, 51), involves an adaptation of the standardized clinical tools (52) and institutions (53). Moreover, theoretical support in recognizing and dealing with transcultural contexts should be developed for personnel, first in their training courses and second by setting up practical guidelines, following examples already existing in the psychiatric field, such as the Cultural Formulation Interview (54, 55). Afterwards, these same school personnel might usefully be offered close psychological supervision or intervention at their workplace. Finally, the use of professional interpreters should be systematized in cases of linguistic barriers.

### Differences Between Paris and Franche-Comté: The French Colonial History

Our study found clear differences between the situations recounted by the participants in Paris and in Bourgogne Franche-Comté. Several factors might explain this contrast. The first is that the context of migration differs substantially between the two areas, with many fewer migrants in Bourgogne Franche-Comté than in the Paris basin (56, 57).

Second, migrant profiles vary notably between these locations. In Bourgogne, the most common young migrants are first-generation political refugees, without their parents, and travelers, the latter now long settled in Bourgogne, with uniquely seasonal moving. Participants' difficulties in talking about the culture of the others appeared more obvious in Paris, where the immigrant youth encountered were mostly from countries that share a history of colonization by France (North Africa and sub-Saharan Africa). This colonial past remains today a very sensitive issue in France, as illustrated by the sparse development of postcolonial studies, compared with other former colonial powers.

Third, institutional support appears to be different. In Bourgogne, participants described substantial support for the refugees from local organizations and the parents of other pupils. For example, school officials often placed them in elite international classes, along with youth from United States and Australia in France for exchange programs.

### Strengths and Limitations

Our study had several strengths. The choice of the subjects interviewed was original and important, because understanding the perspectives and attitudes of school personnel is key to developing interventions that could ensure equality of opportunity to immigrants in schools. Also, the rigorous GT-based analysis was most appropriate to its topic. An important limitation of our study is that it was unable to assess in detail the differences between immigrant families and others. It might have been useful to include school personnel from the more socially disadvantaged suburbs of Paris. This would have allowed us, for example, to give voice to those working in sensitive areas, in “réseaux d'éducation prioritaire (REP)” (high-priority education networks), that is, districts where social difficulties strongly affect academic achievement (58). The school personnel in these districts are more likely to encounter and deal with immigrant families, who often live in these areas. They would not only have had more transcultural contexts to talk about, but would also have been able to make comparisons that would have enabled a more specific focus on the differences between immigrant families and other disadvantaged families.

### Implications

Examination of misconceptions about the absenteeism of children from transcultural backgrounds (migrant children or children from immigrant families) is essential for ensuring equality of opportunity to immigrants in schools in France, Europe, and elsewhere. This research could usefully be extended by including an assessment of transcultural dimensions in the classification of SAPs, especially school refusal. Our results show the need to improve the training of teachers and other school staff to make them aware of students' diversity, to take staff cultural countertransference into account, and to implement transcultural skills in their theoretical training. It also suggests the utility of helping parents to share their efforts to secure their child's attendance with school personnel by promoting the use of professional interpreters during encounters with families with transcultural backgrounds. Finally, these findings also suggest that reflexive practices and creative strategies by school personnel can be supported by setting up close supervision or interventions in the workplace.

## Conclusion

This study describes the ambivalence and the difficulties experienced by school personnel facing school absenteeism of children with transcultural backgrounds. School absenteeism by these children challenges the usual practices of school personnel. When coping with these unusual situations, school personnel are unsettled by families' school cultures, which differ from what they expect. Many participants tried to describe and understand the students' absenteeism without openly addressing their culture. Their misconceptions about these students may lead to misdiagnosis for youths whose school absenteeism might be incorrectly labeled as truancy rather than school refusal. The consideration of parental “reasonable efforts” already takes different parenting styles into account, but continues to overlook cultural factors that can cause the misunderstanding of the youth's behavior and of their parents' attitudes toward school attendance. These feelings reflect the tension between the ideology of a secular Republic and the reality of the school personnel's daily lives, which are becoming increasingly ethnicized. Nonetheless, the creative strategies that school personnel report developing demonstrate that that cultural barriers can be overcome and that unconscious discrimination is not inevitable.

This situation calls for a major change in the understanding of cultural differences in color-blind societies. In the meantime, more local solutions could be attempted. Practices might be improved by developing transcultural theoretical training for professionals working in the area of school absenteeism among students with such backgrounds. Second, in the research field, the assessment of transcultural dimensions should be included in standardized diagnostic tools that aim to differentiate school refusal from other SAPs (truancy, school withdrawal, and school exclusion) to ensure equality of access to services for immigrants in schools, including equality of referral to health care services when needed.

## Data Availability Statement

All datasets generated for this study are included in the article/supplementary material.

## Ethics Statement

The studies involving human participants were reviewed and approved by The Competent Institutional Review Board of the Inserm “Comité d'Evaluation Ethique de l'Inserm (CEEI—IRB00003888). The patients/participants provided their written informed consent to participate in this study.

## Author Contributions

The study was designed by LB, who also asked the competent institutional review board of the Inserm (Comité d'Éthique de l'Inserm) to approve it. All respondents were interviewed by one of the two researchers (RM and LB). Both of them independently coded all interviews. The triangulation of the analysis, which guarantees the quality of individual coding, took place during monthly meetings of our research group (LB, RM, JB, and MM).

## Funding

This study received funding from the French National Institute of Health and Medical Research (Inserm) through LB's PhD grant and from private contributors through the Thellie Foundation for the project “Understanding pathways to care in school refusal and improving them”.

## Conflict of Interest

The authors declare that the research was conducted in the absence of any commercial or financial relationships that could be construed as a potential conflict of interest.
