# Corrigendum: School Refusal or Truancy? A Qualitative Study of Misconceptions Among School Personnel About Absenteeism of Children From Immigrant Families

**DOI:** 10.3389/fpsyt.2020.00300

**Published:** 2020-04-23

**Authors:** Robin Martin, Jean Pierre Benoit, Marie Rose Moro, Laelia Benoit

**Affiliations:** ^1^Maison des Adolescents de Saint-Denis, Saint-Denis Hospital, Saint Denis, France; ^2^Maison des Adolescents–Maison de Solenn, Hôpital Cochin, APHP, Paris, France; ^3^University of Paris, Medical School, Faculty of Psychology, PCPP, Boulogne-Billancourt, France; ^4^Center for Research in Epidemiology and Population Health (CESP), Paris-Sud and UVSQ Medical Schools, French National Institute of Health and Medical Research (Inserm), Team DevPsy, Villejuif, France

**Keywords:** school refusal, truancy, teacher, school personnel, immigrant youth, minorities, discrimination, school absenteeism

In the original article, there was a mistake in the title as published. The first part of the title “School Refusal or Truancy?” was missing in the final version. The complete title appears above. The authors apologize for this error and state that this does not change the scientific conclusions of the article in any way.The original article has been updated.

